# Research Advances on Organelle-Targeted Drug Delivery Systems for the Treatment of Brain Tumors and Central Nervous System Inflammation

**DOI:** 10.3390/pharmaceutics18091140

**Published:** 2026-09-10

**Authors:** Manru Zhang, Bohan Chen, Tiezheng Li, Xiaolong Li, Jingxian Sun, Qiushuo Li, Mingji Jin, Zhonggao Gao, Liqing Chen, Wei Huang

**Affiliations:** 1 State Key Laboratory of Bioactive Substance and Function of Natural Medicines, Institute of Materia Medica, Chinese Academy of Medical Sciences & Peking Union Medical College, Beijing 100050, China; zhangmanru@imm.ac.cn (M.Z.); chenbohan@imm.ac.cn (B.C.); lixiaolong@imm.ac.cn (X.L.); sunjingxian@imm.ac.cn (J.S.); liqiushuo@imm.ac.cn (Q.L.); jinmingji@imm.ac.cn (M.J.); zggao@imm.ac.cn (Z.G.); 2 Beijing Key Laboratory of Key Technologies for Natural Drug Delivery and Novel Formulations, Department of Pharmaceutics, Institute of Materia Medica, Chinese Academy of Medical Sciences & Peking Union Medical College, Beijing 100050, China; 3 Beijing Baheal Chengchuang Pharmaceutical Research and Development Co., Ltd., Beijing 102300, China; litiezheng@baiyyy.com.cn

**Keywords:** central nervous system diseases, suborganelle-targeted delivery, blood–brain barrier, brain tumors, two-stage delivery

## Abstract

Central nervous system (CNS) inflammation and brain tumor treatment are constrained by the heterogeneity of the blood–brain barrier (BBB) and blood–brain tumor barrier (BBTB), as well as by the sequential barriers to drug delivery across lesions, target cells and subcellular organelles. Simply increasing brain exposure does not ensure that drugs reach their actual sites of action. This review systematically examines the pathological roles and therapeutic rationales of mitochondrial, lysosomal, nuclear, endoplasmic reticulum and Golgi apparatus dysfunction in neuroinflammation and glioblastoma within a two-stage delivery framework encompassing barrier crossing, lesion accumulation, cellular uptake and subcellular organelle localization. It also summarizes key design considerations for liposomes, polymeric nanoparticles, biomimetic membrane-based carriers, exosome-like carriers and focused ultrasound-assisted delivery strategies. Furthermore, translational bottlenecks are discussed, including BBB/BBTB heterogeneity, endosomal/lysosomal escape, organelle off-targeting, long-term safety and the extrapolation of preclinical models. Finally, personalized delivery designs guided by cascade targeting, dynamic visualization-based validation and disease stratification are proposed to support precise treatment of CNS inflammation and brain tumors.

## 1. Introduction

Central nervous system (CNS) diseases primarily include neuroinflammatory disorders and brain tumors. Neuroinflammatory disorders can be classified as acute inflammatory diseases, such as ischemic stroke, or chronic inflammatory diseases, exemplified by Alzheimer’s disease. CNS diseases have become major causes of disability and death worldwide. According to the Global Burden of Disease (GBD) 2021 study, the burden of neurological disorders has continued to increase over recent decades. Neurological diseases represented by stroke, Alzheimer’s disease, Parkinson’s disease, and brain tumors collectively cause more than 11 million deaths annually [1]. Although the incidence of brain and other CNS tumors is lower than that of many common solid tumors, their aggressive nature, high recurrence rate, and narrow therapeutic window continue to pose substantial challenges for clinical treatment [2,3]. However, although many candidate drugs exhibit significant pharmacological activity in vitro, their clinical translation remains limited by insufficient brain exposure, heterogeneous distribution within lesions, and inadequate effective intracellular concentrations in target cells [4,5]. These observations indicate that treatment failure in CNS diseases may not solely result from the intrinsic lack of drug efficacy, but may also arise because drugs fail to reach their intended therapeutic targets at sufficient concentrations, with appropriate spatial distribution and correct subcellular localization [6,7,8].

The blood–brain barrier (BBB) constitutes the first barrier to CNS drug delivery. Studies have shown that approximately 98% of small-molecule drugs and nearly 100% of large-molecule biologics fail to achieve effective therapeutic concentrations within the brain parenchyma [9]. The BBB is composed of tight junctions between brain microvascular endothelial cells, the basement membrane, pericytes, astrocytic endfeet, and the neurovascular unit [10]. Together with selective transport systems and efflux transporters such as P-glycoprotein (P-gp) and breast cancer resistance protein (BCRP), it restricts the entry of most small-molecule drugs, macromolecular drugs, nucleic acid therapeutics, and nanomedicines into brain tissue [10,11,12]. In CNS tumors such as glioblastoma, the blood–brain tumor barrier (BBTB) represents the major barrier to drug delivery. The BBTB is often described as a “disrupted BBB”; however, its permeability is not uniform but exhibits marked spatial heterogeneity. Specifically, focal leakage may occur in contrast-enhancing tumor regions, whereas relatively intact barrier function may persist at infiltrative margins and in microscopic residual lesions [13,14]. Therefore, delivery systems that rely solely on the enhanced permeability and retention (EPR) effect or a single BBB-crossing ligand can generally increase drug exposure only in certain lesion regions and are unlikely to adequately cover infiltrative tumor cells, inflammatory border zones, and the deep brain parenchyma [15].

Over the past five years, technologies such as focused ultrasound, receptor-mediated transcytosis, brain-penetrating peptides, cell membrane-biomimetic nanoparticles, exosomes, and lipid nanoparticles have substantially advanced research on delivery across the BBB/BBTB [16]. However, numerous clinical trials have indicated that even when drugs successfully cross the endothelial barrier and enter the brain interstitium, they may still fail to achieve sufficient therapeutic efficacy. For example, Sevigny et al. found that anti-β-amyloid monoclonal antibodies could partially cross the BBB; however, plaque clearance was associated with only modest cognitive improvement in clinical trials of Alzheimer’s disease [17]. In a multicenter phase II clinical trial in patients with recurrent glioblastoma characterized by phosphoinositide 3-kinase (PI3K) pathway activation, Wen et al. found that the pan-PI3K inhibitor buparlisib effectively penetrated tumor tissue and infiltrative brain parenchyma. Nevertheless, the drug did not adequately inhibit PI3K signaling in tumor tissue, and its clinical efficacy as a monotherapy was limited: only 4 of 50 patients achieved progression-free survival at 6 months, and the median progression-free survival was only 1.7 months. These findings indicate that, even after a drug crosses the BBB/BBTB and reaches intracranial lesions, it remains difficult to translate into meaningful clinical efficacy if sufficient and sustained target inhibition cannot be achieved within tumor cells [18].

The second barrier comprises obstacles to intracellular and subcellular organelle delivery after drugs enter the brain parenchyma. The effective targets of many CNS drugs are located not in the extracellular space or on the cell surface, but within mitochondria, lysosomes, the endoplasmic reticulum, the nucleus, or specific intracellular signaling complexes. Therefore, simply increasing the total drug concentration in brain tissue does not ensure delivery to the true site of action [7,19,20]. For example, if nanocarriers are retained within endosomes/lysosomes, RNA therapeutics, protein therapeutics, or some small-molecule drugs may be unable to enter the cytoplasm or target organelles, resulting in apparent delivery success but insufficient actual pharmacological efficacy [21,22,23]. For mitochondria-targeted B-cell lymphoma 2 homology 3 (BH3) mimetics, endosomal retention hinders their effective delivery to the outer mitochondrial membrane, thereby attenuating their ability to induce mitochondrial outer membrane permeabilization [21,24,25]. In neuroinflammatory diseases, assembly of the NOD-, LRR- and pyrin domain-containing protein 3 (NLRP3) inflammasome and regulation of autophagy pathways also depend on precise subcellular localization. Therapeutic molecules must achieve effective exposure in key regions, such as the cytoplasm and lysosomes, to accurately regulate inflammatory signaling and protein homeostasis. Mislocalization during delivery may not only weaken neuroprotective effects, but also increase the risk of off-target immune activation or immunotoxicity [26,27,28]. Therefore, CNS drug delivery should not focus solely on whether a drug can successfully cross the BBB/BBTB and enter the brain. After reaching the brain parenchyma, the drug must further enter target cells and be delivered to the specific subcellular organelles where it exerts its effects. This process may be termed “secondary delivery,” the core of which is precise delivery at the organelle level. It remains constrained by factors such as entrapment in endocytic vesicles, inefficient intracellular transport, and organelle membrane barriers. Therefore, therapeutic molecules can exert their intended effects only when they achieve sufficient, sustained, and precise concentrations in locations such as the cytoplasm, mitochondria, lysosomes, nucleus, or specific signaling complexes. In other words, the efficiency of subcellular organelle delivery is critical in determining whether CNS drugs can progress from “entering the brain” to “producing therapeutic efficacy.”

Inflammatory CNS diseases and CNS tumors cannot simply be classified as a single disease category, nor can they be considered as two entirely unrelated delivery paradigms, as they share certain pathological features. These include dynamic alterations in the BBB/BBTB, immune cell infiltration, increased oxidative stress, dysregulated cytokine networks, and the involvement of phagocytic cells [29,30,31,32]. Therefore, several similar delivery strategies can be applied to both disease categories, including the exploitation of inflammatory chemotaxis, cell membrane camouflage, exosome-mediated intercellular communication, and nanoscale delivery systems responsive to changes in the lesion microenvironment [33,34,35]. Nevertheless, the design priorities differ between the two. For inflammatory CNS diseases, delivery systems place greater emphasis on restoring barrier function at different disease stages, regulating immune-cell phenotypes, and providing neuroprotection [36]. For CNS tumors, design strategies focus more on deep tumor penetration after crossing the BBTB, coverage of infiltrative margins, reversal of the immunosuppressive microenvironment, and elimination of drug-resistant tumor cells [30,32]. For example, in studies of stroke and neurodegenerative diseases, mitochondria-targeted nanosystems have suggested that stepwise “lesion–cell–organelle” delivery can alleviate mitochondrial damage and improve neurological function [37,38]. By contrast, in glioblastoma, mitochondria-targeted or tumor microenvironment-responsive nanomedicines are more commonly used to enhance the targeting of tumor metabolic vulnerabilities and amplify cell-death signaling [39,40].

Accordingly, organelle-targeted treatment is not a single strategy but a disease-context-dependent intervention. In CNS inflammation, mitochondrial targeting is used mainly to restore bioenergetics and limit mitochondrial reactive oxygen species (ROS) production and damage-associated molecular pattern release, thereby reducing microglial activation; lysosomal targeting aims to restore acidification and autophagic clearance while restraining NLRP3 signaling; and interventions directed at the endoplasmic reticulum or nucleus can help attenuate maladaptive stress responses and nuclear factor kappa B (NF-κB) -mediated inflammatory transcription [41,42,43,44,45,46,47,48,49,50]. In CNS tumors, particularly glioblastoma (GBM), the same organelles are exploited differently: mitochondrial targeting disrupts oxidative phosphorylation or amplifies oxidative stress and ferroptotic death, lysosomal manipulation counteracts drug sequestration and protective autophagy or enables endosomal escape, endoplasmic reticulum targeting converts the adaptive unfolded protein response into lethal stress, and nuclear delivery enables gene editing or inhibition of oncogenic and epigenetic programs [51,52,53,54,55,56,57,58,59,60,61,62]. Thus, matching the organelle and payload to the desired direction of functional modulation—protection or homeostatic restoration in inflamed neural cells versus selective dysfunction and death in tumor cells—constitutes the central therapeutic logic of this review.

This review focuses on CNS inflammation and brain tumors and systematically examines the roles of mitochondrial, lysosomal, nuclear, endoplasmic reticulum, and Golgi apparatus dysfunction in disease pathogenesis, therapeutic resistance, and drug responses within a continuous delivery process comprising “BBB/BBTB crossing–lesion accumulation–target-cell uptake–subcellular organelle localization”. Unlike previous reviews that focus solely on brain targeting or localization to a single organelle, this review emphasizes the hierarchical relationship among multilevel barriers in brain tissue, cells, and subcellular organelles, as well as the differences in organelle-targeting rationales between neuroinflammation and glioblastoma. It subsequently summarizes targeted delivery strategies and carrier-design principles for different subcellular organelles and analyzes translational bottlenecks, including BBB/BBTB heterogeneity, endosomal/lysosomal escape, subcellular organelle off-targeting, long-term safety, and extrapolation from preclinical models. Finally, this review discusses personalized delivery strategies driven by multistage cascade targeting, visualization-based validation of delivery, disease stage, and patient characteristics.

## 2. Pathological Associations Between Subcellular Organelle Dysfunction and CNS Diseases

The onset and progression of CNS diseases are associated with dysfunction of multiple subcellular organelles and their interactions [63,64]. Mitochondria, lysosomes, the endoplasmic reticulum, and the nucleus participate in energy supply, intracellular material clearance, protein folding, and regulation of gene transcription, respectively [65]. Dysfunction of these organelles can further trigger pathological processes, including oxidative stress, inflammasome activation, the unfolded protein response (UPR), and epigenetic alterations [66,67,68,69]. From the perspective of pharmaceutical sciences and drug-delivery research, subcellular organelles are not only damaged structures in CNS diseases, but also key sites of action for therapeutic intervention, targeted delivery, and the design of combination therapies.

### 2.1. Mitochondrial Dysfunction

Mitochondria are core organelles that enable neurons and glial cells to maintain adenosine triphosphate (ATP) production, calcium homeostasis, and redox balance. Ionescu-Tucker and Cotman found that oxidative stress levels are markedly elevated during brain aging and Alzheimer’s disease and are closely associated with neuronal injury, impaired synaptic function, and persistent inflammatory responses [41]. These findings suggest that mitochondria-derived oxidative stress is an important driver of neurodegenerative disease progression. Lin et al. further noted that damaged mitochondria can release mitochondrial damage-associated molecular patterns, including mitochondrial DNA, ATP, and ROS. These signals can activate microglia and amplify neuroinflammatory responses, thereby explaining how mitochondrial damage can be translated from disrupted intracellular homeostasis into tissue-level inflammatory pathology [42]. Cheng et al. found that microglia undergo glycolytic reprogramming at an early stage of inflammatory activation, indicating that neuroinflammation is not merely a process of immune mediator release but is also accompanied by alterations in energy metabolism [43]. Wang et al. showed that *Akkermansia muciniphila* and its metabolite propionate can maintain neuronal mitochondrial fission and mitophagy homeostasis through G protein-coupled receptors 41 and 43 (GPR41/GPR43), suggesting that impaired mitochondrial quality control is closely associated with the pathological progression of Alzheimer’s disease [44]. Therefore, in neurodegenerative inflammation, mitochondrial dysfunction is mainly characterized by amplification of oxidative stress and inflammatory signaling. Pharmacological interventions should focus on antioxidant therapy, improvement of mitochondrial quality control, and restoration of energy homeostasis.

Unlike the therapeutic strategy of “protecting mitochondria” in neuroinflammation, mitochondria in glioma often serve as an important metabolic basis for sustaining tumor-cell proliferation, invasion, and stress-resistant survival. Watson et al. found that astrocytes can transfer mitochondria to GBM cells in a growth-associated protein 43 (GAP43) -dependent manner, thereby enhancing tumorigenic potential. This study explained how GBM cells obtain additional metabolic support from the perspective of the tumor microenvironment [51]. Zou et al. developed a mitochondria-targeted nanomedicine loaded with Gboxin and demonstrated that it could inhibit GBM growth by disrupting mitochondrial function, providing experimental evidence for mitochondria-targeted drug delivery in brain tumors [52]. Banu et al. found that certain cellular states of GBM are more sensitive to glutathione peroxidase 4 (GPX4)-dependent ferroptosis, indicating that redox homeostasis and lipid peroxidation may provide important avenues for inducing GBM cell death [53]. Thus, the rationale for mitochondrial targeting differs substantially between CNS inflammation and brain tumors: the former emphasizes functional protection, whereas the latter places greater emphasis on disrupting tumor metabolic dependencies or inducing metabolic cell death.

### 2.2. Lysosomal Dysfunction

Lysosomes are the principal intracellular sites for degradation of proteins, lipids, and damaged organelles, and are also key structures required for the proper progression of autophagy. Li and Gong summarized that the NLRP3 inflammasome contributes to Aβ deposition, cellular injury, and inflammatory cytokine release in Alzheimer’s disease, suggesting a close association between lysosomal damage and inflammasome activation [45]. Huang et al. found that tripartite motif-containing protein 45 (TRIM45) can exacerbate microglial pyroptosis through the autophagy-related protein 5 (Atg5)/NLRP3 axis, indicating that abnormal autophagy regulation directly affects inflammasome activation and promotes inflammatory cell death [46]. Li et al. found that impaired lipophagy leads to the accumulation of lipid droplets and triggers receptor expressed on myeloid cells 1 (TREM1) in microglia, thereby promoting inflammatory responses in diabetes-associated cognitive impairment. This finding indicates that lysosomal dysfunction can also participate in CNS inflammation by disrupting lipid metabolism [47].

In brain tumors, lysosomes and the autophagy system more commonly function as adaptive protective mechanisms that enable tumor cells to withstand therapeutic stress. Hwang et al. found that temozolomide (TMZ) treatment can induce protective autophagy in GBM cells and that abnormal autophagy regulation is closely associated with TMZ resistance. This result indicates that DNA alkylation damage alone may not be sufficient to completely eliminate GBM cells [54]. Li et al. systematically summarized the molecular mechanisms underlying TMZ resistance in GBM, noting that, in addition to O6-methylguanine-DNA methyltransferase (MGMT) -mediated DNA repair, enhanced autophagy, adaptation to oxidative stress, metabolic remodeling, and evasion of cell death also contribute to the development of resistance [55]. From disease and therapeutic perspectives, Zhang et al. noted that lysosomes not only participate in intracellular degradation but are also closely associated with drug accumulation, cell death, and therapeutic responses. This provides a theoretical basis for understanding lysosomal sequestration and drug resistance in tumors [56]. Therefore, the pathological significance of lysosomes in neuroinflammation lies in impaired clearance and inflammasome activation, whereas in GBM it is mainly reflected in protective autophagy and therapeutic resistance.

### 2.3. Endoplasmic Reticulum Dysfunction

The endoplasmic reticulum is responsible for protein folding, maintenance of calcium homeostasis, and lipid synthesis. Its dysfunction can induce the unfolded protein response (UPR) and affect cell survival or death through branches including protein kinase R-like endoplasmic reticulum kinase (PERK), inositol-requiring enzyme 1 (IRE1), and activating transcription factor 6 (ATF6). Liu et al. summarized that endoplasmic reticulum stress is widely involved in multiple disease processes and represents an important link between disrupted proteostasis, oxidative stress, and cell death [70]. Kou et al. found that flavone could ameliorate cognitive impairment in mice with Alzheimer’s disease by inhibiting endoplasmic reticulum stress-dependent neuroinflammation, suggesting that endoplasmic reticulum stress (ER stress) is an intervenable pathological process in neuroinflammation [48]. Wang et al. found that saikosaponin B2 could attenuate microglial activation in a depression model by inhibiting ferroptosis-related neuroinflammation and ER stress, indicating a mutually reinforcing relationship among ER stress, oxidative damage, ferroptosis, and inflammatory responses [71]. Therefore, in inflammatory CNS diseases, ER stress mainly amplifies stress signaling and can transform proteostasis imbalance into persistent inflammatory responses.

In GBM, the UPR has a clear dual role. Moderate UPR activation can help tumor cells adapt to hypoxia, nutrient deprivation, and therapeutic stress, whereas excessive ER stress may induce tumor-cell death. Zielke et al. found that activating transcription factor 4 (ATF4) links ER stress to ER-phagy, enabling GBM cells to clear damaged endoplasmic reticulum and maintain survival. This finding indicates that tumor cells can adapt to persistent stress through organelle quality control [57]. Ketkar et al. found that inhibition of PERK-mediated UPR could reverse the senescent state of residual GBM cells after treatment and induce a senolytic effect, suggesting that the PERK pathway is a potential target for eliminating residual tumor cells after therapy [58]. Lee et al. found that migrasome-autophagosomes could alleviate ER stress in GBM cells, indicating that GBM cells can use noncanonical autophagic structures to manage organelle stress and maintain survival [59]. Thus, ER stress mainly acts as a damage amplifier in neuroinflammation, whereas in GBM it has dual significance: it can help tumor cells adapt to stress and sustain survival, but it can also be further amplified pharmacologically and converted into a therapeutic vulnerability that induces tumor-cell death.

### 2.4. Nuclear Dysfunction

The nucleus is the core structure responsible for maintaining genetic information and regulating gene transcription. Its abnormalities are mainly manifested as changes in chromatin architecture, epigenetic reprogramming, and aberrant activation of inflammation-related transcription factors. Chaligne et al. found that glioma transcriptional cell states are epigenetically encoded, heritable, and plastic, indicating that GBM heterogeneity is not determined solely by genetic mutations but is also influenced by dynamic changes in chromatin states [60]. Sun et al. found that bromodomain-containing protein 8 (BRD8) can epigenetically remodel the p53 network to sustain GBM growth, suggesting that chromatin-regulatory proteins may serve as important targets for therapeutic intervention in glioma [61]. De Leo et al. found that glucose-driven histone lactylation can enhance the immunosuppressive activity of monocyte-derived macrophages in GBM, indicating that metabolites can also reshape the tumor immune microenvironment through nuclear epigenetic modifications [62]. These studies suggest that nuclear abnormalities in brain tumors are not only characterized by increased expression of proliferation-related genes, but also by coordinated alterations in metabolism, epigenetic regulation, and the immune microenvironment.

In neuroinflammation, nuclear dysfunction is more commonly characterized by the nuclear translocation of inflammation-related transcription factors, such as NF-κB, and the subsequent upregulation of inflammatory gene expression. Mockenhaupt et al. noted that RelB, as a component of the noncanonical NF-κB pathway, participates in the regulation of neuroinflammation, indicating that the NF-κB family has multilayered regulatory roles in CNS inflammation [49]. Liu et al. found that astrocyte-derived SerpinA3N can promote neuroinflammation and epileptic seizures by activating NF-κB signaling, indicating that nuclear inflammatory transcriptional programs in glial cells can directly affect the manifestations of CNS diseases [50].

In summary, the pathological significance of subcellular organelle dysfunction in CNS diseases is clearly disease-context-dependent. At the mitochondrial level, neuroinflammation emphasizes the amplification of oxidative injury, whereas GBM emphasizes metabolic dependence. At the lysosomal level, neuroinflammation emphasizes impaired clearance and NLRP3 inflammasome activation, whereas GBM emphasizes protective autophagy and therapeutic resistance. At the endoplasmic reticulum level, neuroinflammation emphasizes ER stress-mediated amplification of inflammatory signaling, whereas GBM emphasizes UPR-mediated adaptive survival. At the nuclear level, neuroinflammation emphasizes NF-κB-driven inflammatory transcriptional programs, whereas GBM emphasizes chromatin remodeling and epigenetic plasticity. Collectively, these mechanisms indicate that subcellular organelles are not only pathologically damaged structures in CNS diseases, but also precision targets with translational potential for drug development and drug-delivery system design.

## 3. Subcellular Organelle Targeting Strategies and Carrier Design

The preceding sections systematically reviewed the pathological alterations and mechanisms of functional dysregulation of mitochondria, lysosomes, the endoplasmic reticulum, and the nucleus in CNS inflammation and glioblastoma. However, translating these pathological features into effective pharmacological interventions requires addressing a central delivery challenge: how can therapeutic molecules, after crossing the BBB/BBTB, further achieve secondary delivery and attain effective concentrations within the appropriate organelles? The following sections summarize, for each organelle, the rationale for targeting, representative carrier strategies, and their applicable boundaries in inflammatory and tumor settings. On this basis, potential trade-offs among different targeting requirements are also discussed.

### 3.1. Representative Carrier Platforms for Brain-Targeted and Organelle-Targeted Delivery

Carrier architecture determines payload protection, pharmacokinetic behavior, interaction with the BBB/BBTB, and intracellular trafficking after brain entry. The liposomal, polymeric, biomimetic membrane-based, and exosome-like platforms discussed in this review therefore provide complementary rather than interchangeable delivery functions. Their principal characteristics and representative applications are briefly summarized below.

Liposomes are phospholipid-bilayer vesicles that can encapsulate hydrophilic agents in an aqueous core and hydrophobic agents within the lipid membrane. Their composition and surface can be modified with polymers, targeting ligands, or stimuli-responsive groups to regulate circulation, BBB interaction, and intracellular release. For example, glucose- and triphenylphosphonium (TPP^+^) co-modified redox-responsive liposomes were used to deliver doxorubicin and lonidamine to glioma. Glucose promoted uptake by brain tumor-associated cells, whereas TPP^+^ enhanced mitochondrial accumulation [72]. Polymeric nanoparticles, in contrast, are assembled from natural or synthetic polymers and offer tunable particle size, surface chemistry, mechanical stability, and drug-release kinetics. Angiopep-2-modified lipid-polymer nanoparticles provide a representative example: they crossed an experimental BBB and delivered clustered regularly interspaced short palindromic repeats-associated protein 9 (Cas9)/single-guide RNA (sgRNA) to GBM, where functional signal transducer and activator of transcription 3 (STAT3) editing remodeled the vascular and immune microenvironment [73]. The main translational constraints of these synthetic platforms include premature payload release, uptake by peripheral phagocytes, incomplete endosomal escape, material-associated toxicity, and reproducibility during scale-up [14,15,16,74,75].

Biomimetic membrane-based carriers generally consist of a synthetic therapeutic core coated with cell-derived or organelle-derived membranes. The retained membrane proteins can confer immune evasion, inflammatory tropism, homologous tumor recognition, or subcellular targeting. In a Parkinson’s disease model, TPP-modified macrophage membranes were used to coat Cu_2−_xSe/curcumin nanoparticles, supporting inflammatory lesion recognition and subsequent delivery to neuronal mitochondria [76]. In GBM, a Gboxin nanomedicine coated with hybrid cancer-cell and mitochondrial membranes combined homologous tumor-cell uptake with mitochondrial localization [52]. Exosome-like carriers are nanoscale vesicular systems that reproduce selected features of natural extracellular vesicles, including a lipid membrane, membrane proteins, and the capacity for intercellular cargo transfer. Macrophage-derived nanovesicles carrying ursodeoxycholic acid exploited inflammatory tropism to cross the BBB and deliver the drug to damaged neuronal mitochondria [77]. Other examples include Angiopep-2/transactivator of transcription (TAT) -engineered small extracellular vesicles for sequential BBB crossing and glioma-cell uptake [78], and neutrophil exosomes that respond to the inflammatory GBM microenvironment [79]. However, source-cell heterogeneity, variable membrane composition, cargo-loading efficiency, product purity, storage stability, and batch-to-batch consistency remain important manufacturing and quality-control challenges for both biomimetic and exosome-like systems [80,81,82].

Accordingly, no carrier class is universally superior. Liposomes and polymeric nanoparticles offer relatively controllable formulation and release properties, whereas membrane-based and exosome-like carriers can provide more biologically derived recognition functions. Platform selection should therefore be guided by payload type, administration route, BBB or BBTB status, target-cell identity, intended organelle, dosing frequency, and the feasibility of reproducible manufacturing.

### 3.2. Mitochondrial Targeting

The rationale for mitochondrial targeting mainly arises from the strong negative membrane potential of the inner mitochondrial membrane and the central roles of mitochondria in oxidative phosphorylation, ROS generation, calcium homeostasis, apoptosis, and metabolic reprogramming [83,84]. TPP^+^ is a commonly used mitochondrial-targeting moiety. Its lipophilic and cationic properties facilitate the membrane potential-driven accumulation of drugs or nanocarriers in mitochondria [72,85]. For example, redox-responsive liposomes co-modified with glucose and TPP^+^ can enhance uptake by brain tumor-associated cells through glucose and increase mitochondrial uptake within glioma cells through TPP^+^, thereby achieving synergistic anti-glioma effects of doxorubicin and lonidamine [72].

In recent years, membrane-biomimetic strategies have provided new approaches for designing mitochondria-targeted nanomedicines. In a Parkinson’s disease model, Cu_2−_xSe/curcumin nanoparticles coated with TPP-modified macrophage membranes could respond to the inflammatory microenvironment in the brain and subsequently localize to mitochondria in damaged neurons, thereby promoting mitochondrial biogenesis through nicotinamide adenine dinucleotide (NAD^+^)/sirtuin 1 (SIRT1)/peroxisome proliferator-activated receptor gamma coactivator 1-alpha (PGC-1α) -related pathways [76]. Macrophage-derived nanovesicles carrying ursodeoxycholic acid can cross the BBB by exploiting inflammatory tropism and deliver the drug to damaged neuronal mitochondria, thereby improving mitochondrial dysfunction in neurodegenerative diseases [77]. In cerebral ischemia–reperfusion injury, polyoxometalate nanomedicines modified with tannic acid and melanin can recognize matrix proteins exposed on the damaged BBB and subsequently target outer mitochondrial membrane proteins in neurons, achieving sequential targeting from the lesioned brain region to neuronal mitochondria [86]. These studies indicate that mitochondria-targeted delivery no longer relies solely on membrane potential-driven accumulation, but is gradually evolving into a hierarchical delivery mode comprising “lesion recognition–cell-type selection–mitochondrial localization”.

**Figure 1:**
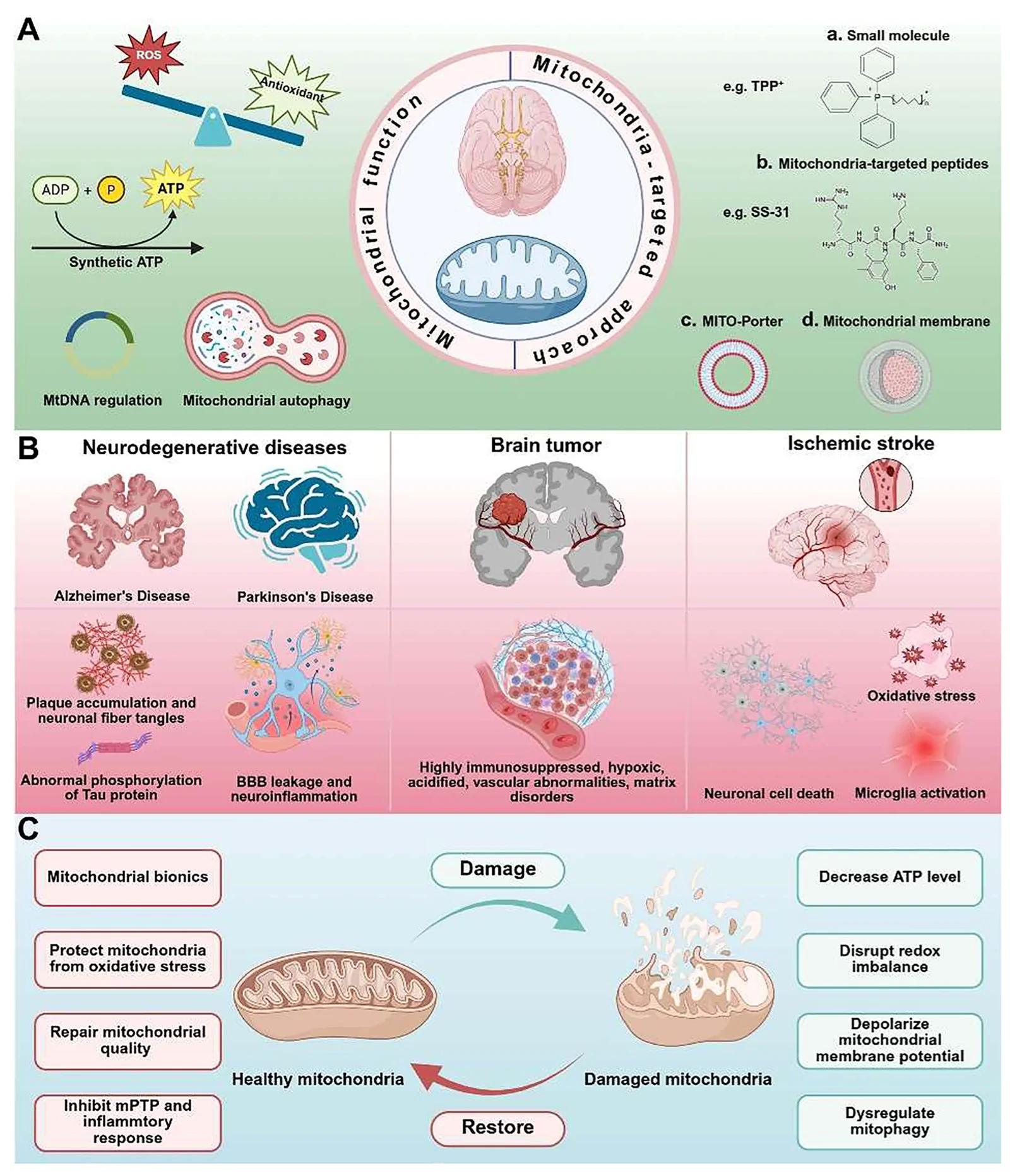
Schematic illustration of mitochondria-targeted nanosystems in CNS disorders. (**A**) Mitochondrial function and mitochondria-targeted approaches. (**B**) The microenvironment of mitochondria-related CNS diseases. (**C**) The role of mitochondria-targeted nanosystems in regulating the mitochondria function. Reproduced with permission [89]. This is an open access article distributed under the terms of the Creative Commons CC BY license.

In glioblastoma therapy, mitochondrial targeting is often combined with metabolic inhibition, ferroptosis, cuproptosis, and photodynamic therapy. For example, a Gboxin nanomedicine coated with hybrid cancer cell and mitochondrial membranes can increase BBB permeability, enhance uptake by homologous tumor cells and mitochondrial localization, and exert anti-GBM effects by inhibiting oxidative phosphorylation [52]. Angiopep-2-modified, macrophage membrane-camouflaged nanoparticles can cross the BBB to deliver small activating RNA targeting arachidonate 15-lipoxygenase (saALOX15) and enhance the radiosensitivity of GBM by inducing mitochondrial damage-associated ferroptosis [87]. A brain-targeted nanoassembly constructed from triphenylphosphonium-chlorin e6 (TPP-Ce6) and copper ions can promote ROS accumulation in mitochondria and reshape the tumor immune microenvironment through cuproptosis and the cyclic GMP-AMP synthase-stimulator of interferon genes (cGAS-STING) pathway [88]. Overall, mitochondrial targeting has accumulated substantial experimental support in mouse models of brain diseases; however, the stability of its subcellular localization in vivo, its long-term safety, and its potential for genuine clinical translation still require further validation (Figure 1).

### 3.3. Lysosomal Targeting

For siRNA, mRNA, proteins, or gene-editing systems, escape from the endosomal/lysosomal system and entry into the cytoplasm are generally required before they can exert their effects [90,91,92,93,94]. In contrast, for strategies involving protein aggregate clearance, restoration of lysosomal acidification, or enzyme replacement therapy, drugs or carriers need to be delivered more efficiently to lysosomes and retained there for a certain period [95,96,97]. Therefore, lysosomal targeting should not be simply understood as “promoting escape”, but should be classified according to the actual site of drug action into “lysosomal escape-type carriers” and “lysosome-delivery-type carriers”.

For nucleic acid delivery, high cellular uptake does not necessarily indicate high delivery efficiency, because most RNA payloads become trapped in endosomes or lysosomes and only a small fraction can enter the cytoplasm to exert their effects. Nanoscale imaging studies have shown that productive mRNA escape is more likely to occur in early endosomes or recycling endosomes, whereas prolonged endocytic retention may lead to abnormal endosomal acidification and unproductive delivery [92]. Further studies have indicated that, during lipid nanoparticle (LNP) delivery, ionizable lipids and RNA may become spatially separated during endosomal sorting. Even when the endosomal membrane is disrupted, functional RNA payloads may not necessarily be released [98]. Therefore, simply increasing cellular endocytic uptake is insufficient to optimize RNA nanodelivery; post-endocytic escape efficiency and payload integrity are the key determinants of pharmacological activity.

To address this bottleneck, recent material-design strategies have mainly focused on mild acidic pH responsiveness, exposure of positive charges upon protonation, fluorination, and replacement of hydrophilic polymers. For example, the engineered truncated H-ferritin nanocarrier with a positively charged cavity [tHFn(+)] can exploit the BBB-crossing and GBM-targeting capabilities of ferritin, disassemble in the mildly acidic endosomal environment, and expose internal positive charges, thereby promoting siRNA escape from lysosomes [99]. Hybrid-targeted nanoparticles constructed with a docosahexaenoic acid (DHA) -temozolomide lipid core can promote lysosomal escape through lipid peroxidation, improve TMZ delivery efficiency in GBM, and enhance its antimigratory effects [100]. In addition, replacing poly(ethylene glycol) (PEG) lipids with poly(carboxybetaine) lipids can enhance endosomal escape of mRNA-LNPs and reduce immunogenicity, indicating a potential trade-off between conventional PEG-based stabilization strategies and intracellular release efficiency [101]. Incorporation of a small amount of fluorinated poly(ethylene glycol)-1,2-distearoyl-sn-glycero-3-phosphoethanolamine (PEG-DSPE) into LNPs can significantly enhance mRNA expression, which is associated with enhanced membrane interactions and improved endosomal escape [102].

**Figure 2:**
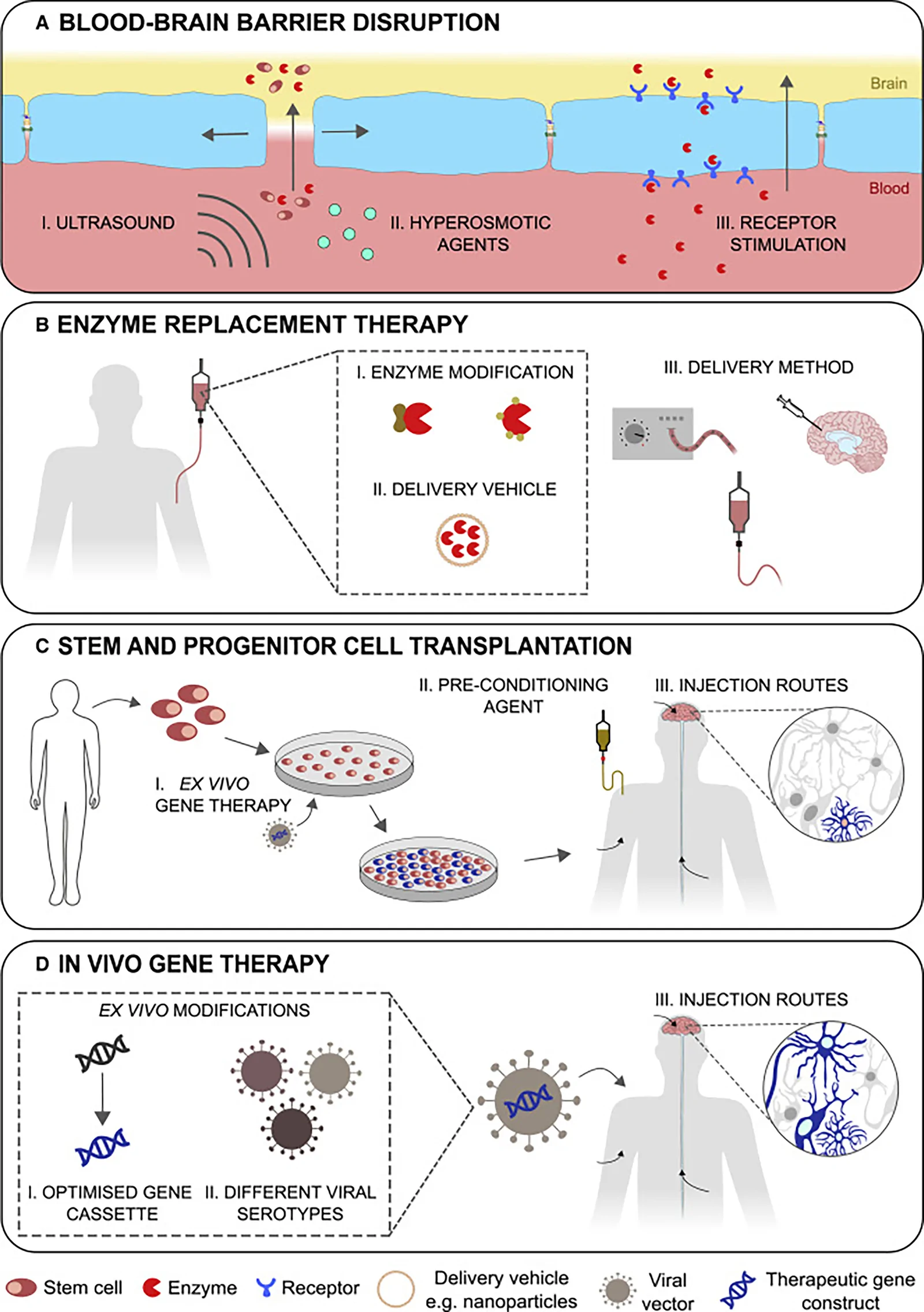
Summary of the different strategies used to improve delivery of therapeutic agents to the CNS in the treatment of LSDs. (**A**) Blood–brain barrier (BBB) disruption strategies: (I) ultrasound or (II) hyperosmotic agents can be used to disrupt the integrity of the BBB; (III) stimulation of receptors can increase passage of enzymes and/or stem cells across the BBB. (**B**) Enzyme replacement therapy can be targeted to the CNS by modifying enzymes directly (I) or using delivery vehicles to facilitate easier passage across the BBB (II). A range of delivery methods (III) including convection-enhanced delivery, direct injection routes, and delivery vehicles can be used to target the CNS. (**C**) Ex vivo genetic modification of stem cells using gene therapy (I), different pre-conditioning regimes/agents (II), and different injection routes (III) have been trialled to improve CNS targeting in stem and progenitor cell transplantation. (**D**) Modifications of gene therapy constructs, including optimization of the gene cassette (I) and selection of viral serotype with CNS tropism (II), and specific injection routes (III) can be utilized to target the CNS with in vivo gene therapy approaches. Reproduced with permission [107]. Copyright {2023}, Cell Press.

However, enhancing endosomal/lysosomal escape is not without risk. For example, LNP-induced endosomal membrane damage can be recognized by galectins and trigger inflammatory responses. This indicates that the design of lysosomal escape-capable carriers should balance release efficiency against membrane damage-related toxicity, rather than simply pursuing stronger membrane-disruptive capacity [103]. In contrast, lysosomes themselves are often therapeutic targets in neurodegenerative diseases. Defective lysosomal acidification is considered one of the early pathological events in Alzheimer’s disease and Parkinson’s disease. Vacuolar-type H+-adenosine triphosphatase (V-ATPase), ion channels, and lysosomal pH homeostasis can all affect the clearance of aberrant proteins [104,105]. Lysosome-shuttle nanochimeras (endoTACs) can promote the endocytosis and lysosomal degradation of receptor for advanced glycation end products (RAGE) through multivalent binding, providing a design strategy for the clearance of aberrant membrane proteins in Alzheimer’s disease based on “delivery to lysosomes” rather than “escape from lysosomes” [106] (Figure 2).

### 3.4. Nuclear Targeting

Nuclear-targeted delivery is mainly used for DNA therapeutics, gene-editing systems, and antitumor drugs that act on DNA or transcriptional regulatory processes. The major barriers are the nuclear envelope and nuclear pore complexes. Commonly used strategies include surface modification of carriers with nuclear localization signals (NLSs) to enable importin-mediated active transport into the nucleus, or optimization of carrier size, charge, and the site of intracellular release to increase the likelihood of drug or gene entry into the nucleus [108,109]. Studies of tumor nanodelivery have shown that nuclear targeting can increase the local concentration of drugs near targets related to DNA, topoisomerases, or transcriptional regulation, thereby enhancing antitumor effects and, to some extent, reducing off-target effects in the cytoplasm [108].

**Figure 3:**
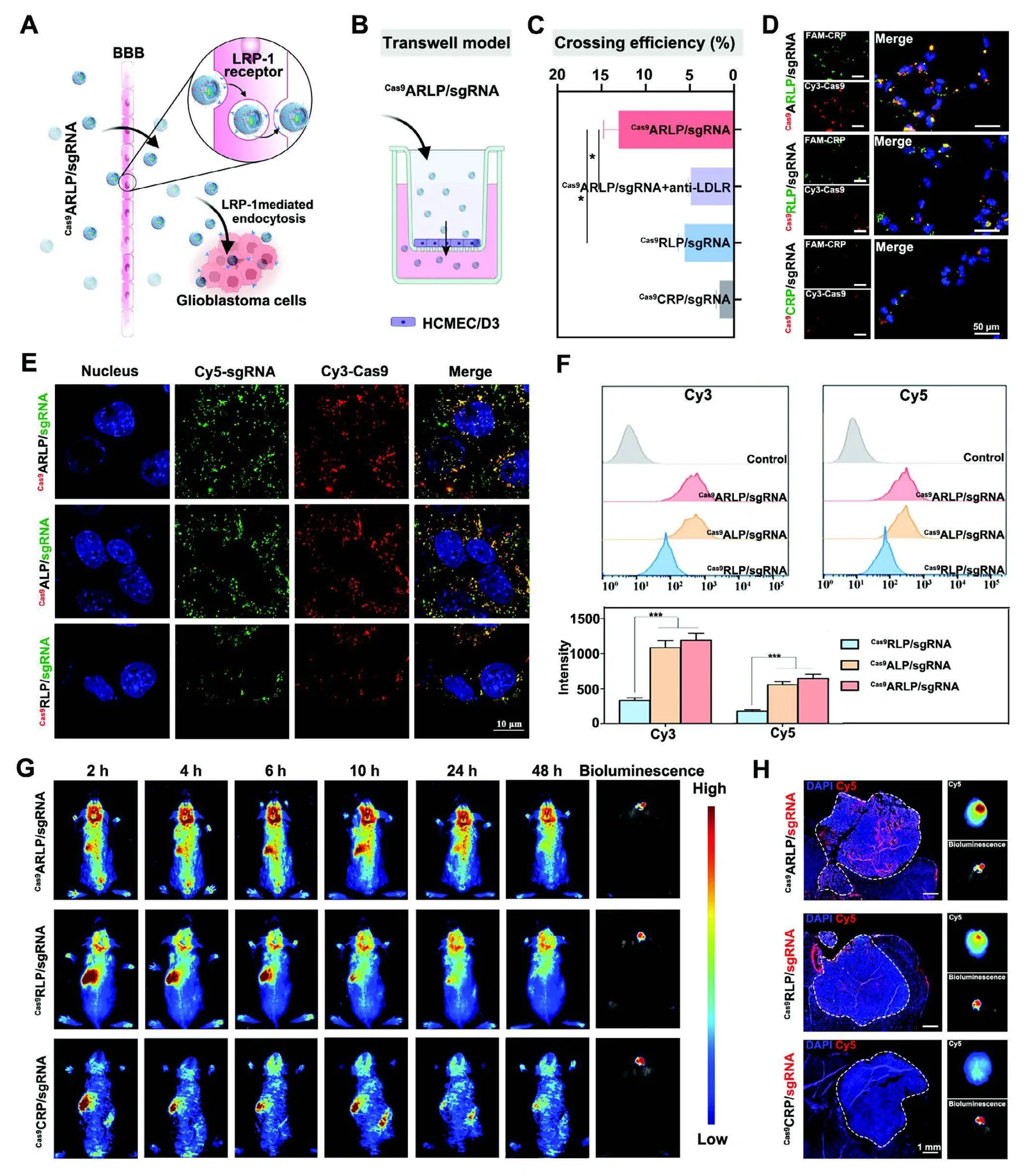
Blood–brain barrier (BBB) crossing delivery of Cas9 and sgRNA to glioblastoma. (**A**) Schematic illustration of Cas9ARLP/sgRNA nanoparticlemediated BBB crossing. (**B**) Schematic illustration of the in vitro BBB model. (**C**) BBB crossing efficiency of the Cas9ARLP/sgRNA, Cas9RLP/sgRNA, Cas9CRP/sgRNA and Cas9ARLP/sgRNA+anti-LDLR groups (*n* = 3). HCMEC/D3 cells were pretreated with anti-LDLR in the Cas9ARLP/sgRNA+antiLDLR group to occupy LDLR before incubation with Cas9ARLP/sgRNA nanoparticles. (**D**) GL261 cell uptake of Cas9ARLP/sgRNA, Cas9RLP/sgRNA, and Cas9CRP/sgRNA after crossing the hCMEC/D3-BBB monolayer model. (**E**) The intracellular distribution of Cas9 and sgRNA mediated by nanoparticles of Cas9ARLP/sgRNA, Cas9ALP/sgRNA, and Cas9RLP/sgRNA. (**F**) Flow cytometry analysis of Cas9 and sgRNA uptake mediated by Cas9ARLP/sgRNA, Cas9ALP/sgRNA, and Cas9RLP/sgRNA nanoparticles (*n* = 3). (**G**) In vivo biodistribution of nanoparticles imaged under the Cy5 channel. (**H**) Ex vivo fluorescence images of brains and colocalization analysis of tumors with nanoparticles. Data are given as means ± SD, * *p* < 0.05, *** *p* < 0.001. Reproduced with permission [73]. This is an open access article distributed under the terms of the Creative Commons CC BY license.

However, in CNS delivery, studies directly demonstrating NLS–nuclear pore complex-mediated nuclear localization in vivo remain markedly fewer than those focused on mitochondria or lysosomes. Current studies more commonly infer delivery efficacy indirectly from functional outcomes, such as gene editing after Cas9/sgRNA delivery, protein expression after mRNA delivery, or changes in nuclear target-gene expression. Angiopep-2-modified lipid–polymer nanoparticles can cross the BBB to deliver Cas9 and sgRNA and remodel the vascular and immune microenvironment of GBM through STAT3 knockout (Figure 3). Functionally, these results suggest that the payload ultimately enters a nuclear compartment in which gene editing can occur [73]. Lipid nanoparticles (LNPs) constructed with BBB-crossing lipids can transfect neurons and astrocytes throughout the brain after systemic administration, indicating progress in intracerebral nucleic acid delivery at the tissue and cellular levels. However, because mRNA mainly acts in the cytoplasm, this does not equate to classical nuclear targeting [110]. Similarly, OS4T LNPs incorporating a 5-hydroxytryptamine type 3 (5-HT3) receptor ligand can substantially improve intracerebral mRNA translation efficiency and are more appropriately discussed in terms of post-BBB cellular uptake and cytoplasmic expression rather than classical nuclear targeting [111]. In addition, glucose transporter 1 (GLUT1) monoligand dual-targeting LNPs can exploit GLUT1 expression in BBB endothelial cells and GBM cells to achieve sequential targeting across the BBB and tumor-cell uptake, providing a design concept in which a single ligand enables dual-layer targeting [106]. Therefore, caution is required when discussing nuclear targeting: although current CNS nanoplatforms have achieved robust functional nucleic acid delivery, direct nuclear localization in vivo still requires further confirmation using approaches such as real-time imaging, nuclear fractionation, spatial omics, or single-particle tracking.

### 3.5. Endoplasmic Reticulum, Golgi Apparatus, and Other Emerging Targets

Compared with mitochondria, lysosomes, and the nucleus, research on endoplasmic reticulum- and Golgi-targeted nanodelivery in brain diseases remains at an early stage. The main challenge is that the endoplasmic reticulum and Golgi apparatus are dynamic endomembrane systems. After entering cells, nanocarriers need to distinguish relatively precisely among endocytic vesicles, regions of endoplasmic reticulum stress, Golgi trafficking pathways, and exosome biogenesis pathways. Although current colocalization imaging methods can provide some clues, they are generally insufficient to fully demonstrate that carriers have achieved genuine functional delivery [112]. From a pharmaceutical perspective, the value of targeting these organelles lies in regulating protein folding, lipid metabolism, vesicular transport, and cellular stress responses. However, carrier design still lacks relatively mature, generalizable approaches comparable to TPP^+^, NLSs, or pH-responsive materials [113].

Overall, different subcellular organelles impose distinct requirements on the physicochemical properties of nanocarriers, and these requirements may constrain one another. Mitochondrial targeting generally requires lipophilic cations or biomimetic membrane recognition, whereas excessively strong positive charges may increase nonspecific membrane toxicity. Lysosomal escape requires protonatable, membrane-disruptive, or membrane-perturbing materials, but excessive endosomal damage can trigger inflammatory responses. Nuclear delivery requires payloads to overcome restrictions imposed by the nuclear envelope and nuclear pore complexes; however, in CNS delivery studies, many investigations still rely mainly on functional outcomes, and direct evidence of nuclear localization remains relatively limited. Therefore, crossing the BBB should be regarded only as the first step in brain-targeted delivery. More convincing future studies should further demonstrate that carriers can sequentially reach the target brain region, target cell type, and target organelle, and should combine approaches such as in vivo imaging, organelle isolation, spatial omics, or single-cell multiomics to establish clearer causal relationships between the delivery process and pharmacological outcomes (Table 1).

## 4. Differences in Brain-Targeted Delivery Logic Between Inflammation and Tumors

Inflammatory brain diseases and brain tumors are both affected by the BBB and its pathological alterations. However, their differences extend beyond variations in barrier permeability and also involve the pathological microenvironment, target-cell types, therapeutic objectives, and administration strategies. In inflammatory diseases, BBB dysfunction usually manifests as a dynamic, stage-dependent, and, to some extent, reversible increase in permeability. Therefore, delivery systems should capitalize on this pathological window to achieve inflammation regulation, neuroprotection, and restoration of BBB function [114,115,116]. In contrast, the BBTB in GBM exhibits marked spatial heterogeneity: barrier integrity differs among the tumor core, infiltrative margins, and postoperative residual lesions, such that passive leakage or the EPR effect alone often cannot yield stable therapeutic outcomes [14,117,118]. Therefore, evaluation of brain-delivery systems should not focus solely on whether they can cross the BBB, but should further assess whether they can precisely reach target cells within specific pathological microenvironments and exert therapeutic effects at appropriate subcellular locations in accordance with disease biology.

### 4.1. Differences in the Pathological Microenvironment

In neuroinflammation, increased BBB permeability is usually associated with disrupted tight junctions between brain microvascular endothelial cells, pericyte dysfunction, alterations in astrocytic endfoot structure, and infiltration of peripheral immune cells [114,115,116]. After stroke, BBB injury and neuroinflammation can mutually reinforce each other, leading to a progressively worsening process. Meanwhile, the degree of barrier opening changes across distinct stages, including acute injury, subacute repair, and chronic inflammation, indicating that the drug-delivery window in inflammatory diseases is strongly time-dependent [114]. In neuroimmune diseases, the degree of BBB disruption is also closely related to disease activity. On the one hand, barrier opening may facilitate drug entry into the brain parenchyma; on the other hand, it may promote the migration of inflammatory cells into the brain, thereby aggravating local immune responses [115]. In addition, studies of persistent systemic inflammation indicate that the BBB in chronic inflammatory states is not a structure that remains stably open, but is dynamically regulated by the level of peripheral inflammation and the state of the neurovascular unit [119]. Therefore, delivery strategies for inflammatory brain diseases should place greater emphasis on controllability, mildness, and reversibility, rather than merely increasing drug exposure in the brain.

The BBTB in brain tumors exhibits more complex structural and functional characteristics. Single-cell studies have revealed substantial differences between the glioma BBTB and the normal BBB in endothelial cell states, transport pathways, and intercellular interactions. In the tumor core of GBM, blood vessels are often abnormal and relatively more permeable. However, barrier function may remain largely intact at infiltrative tumor margins and in microscopic residual lesions. This interregional heterogeneity is one of the major reasons for the inconsistent performance of nanomedicine delivery in brain tumors [14,118]. Therefore, drug delivery for brain tumors generally requires the integration of strategies such as active targeting, receptor-mediated transcytosis, transient focused ultrasound-mediated BBB opening, convection-enhanced delivery, or postoperative local sustained-release approaches to mitigate insufficient drug distribution resulting from BBTB heterogeneity.

### 4.2. Differences in Target-Cell Types

The main delivery target cells in inflammatory brain diseases include microglia, astrocytes, brain microvascular endothelial cells, and immune cells that infiltrate the brain. Microglia are key regulators of neuroinflammation, and their polarization state, phagocytic function, inflammasome activation, and lysosomal function all affect disease progression [120,121]. Studies have shown that microglia-targeted mRNA nanoparticles can ameliorate BBB injury after ischemic stroke by modulating microglial polarization. This finding suggests that the aim of inflammatory delivery is not simply to eliminate relevant cells, but to regulate their abnormal activation and restore relatively normal function [122]. Astrocytes also contribute to inflammation-associated BBB injury. After hemorrhagic stroke, certain inflammation-responsive astrocyte subtypes can directly damage the BBB, indicating that inhibition of harmful glial responses may help maintain neurovascular unit stability [123]. In addition, peripheral inflammation can further aggravate BBB injury by activating the NLRP3–gasdermin D pathway in microglia and promoting neutrophil chemotaxis. Therefore, delivery design for inflammatory brain diseases should simultaneously consider the interactions between glial cells in the brain and peripheral immune cells [124].

Delivery targets in brain tumors place greater emphasis on eliminating or inhibiting tumor-associated cells. In addition to glioma cells, the major targets include glioma stem-like cells, tumor-associated macrophages and microglia, myeloid-derived suppressor cells, and cells associated with abnormal vasculature [118,125]. GBM invasiveness, recurrence, and drug resistance are closely associated with glioma stem-like cells. Therefore, delivery systems must not only cross the BBTB, but also possess effective tumor tissue penetration and improve intracellular drug-release efficiency [125]. For example, Angiopep-2/TAT bifunctionalized engineered small extracellular vesicles can simultaneously promote BBB crossing and glioma-cell uptake, demonstrating the importance of combining a “barrier-crossing ligand” with a ligand that promotes cellular endocytosis in brain-tumor delivery [78]. In addition, neutrophil exosomes and neutrophil-mediated delivery strategies can exploit the inflammatory tumor microenvironment to target GBM. This indicates that inflammatory signals in tumors are not only pathological factors requiring control, but can also be transformed into signals that assist delivery-system localization to tumors [79,126].

### 4.3. Differences in Therapeutic Objectives

Drug delivery in inflammatory diseases generally aims to “modulate, protect, and clear”, including suppression of excessive inflammatory responses, improvement of mitochondrial function, promotion of autophagy–lysosome-mediated clearance of aberrant materials, alleviation of oxidative stress, and repair of the neurovascular unit. Impaired lysosomal acidification in microglia is considered an important mechanism in neuroinflammation and neurodegenerative diseases. Therefore, restoration of lysosomal function represents an important direction for subcellular targeting in inflammatory delivery [121]. In a Parkinson’s disease model, biomimetic targeted nanoparticles exerted therapeutic effects by improving neuronal mitochondrial function and promoting mitochondrial biogenesis, indicating that mitochondria are more suitable targets for functional protection in inflammatory and neurodegenerative diseases [76]. Similarly, mitochondria-targeted nanovesicles delivering ursodeoxycholic acid can improve mitochondrial function in neurodegenerative injury, further supporting the value of mitochondria-protective delivery strategies [77]. In addition, dopamine-conjugated extracellular vesicles can induce autophagy and improve Parkinson’s disease-associated pathology, suggesting that promotion of pathological protein clearance is also an important therapeutic endpoint in inflammatory disease delivery [127]. Cerium oxide nanoparticles can ameliorate ischemic stroke through antioxidant and neurorepair-promoting effects, reflecting the fundamental inflammatory-delivery concept of “reducing injury and promoting repair” [128].

Unlike inflammatory diseases, the primary goals of brain-tumor delivery are to kill tumor cells, inhibit their proliferation, and reverse drug resistance as far as possible. Delivery systems for GBM generally need to induce apoptosis, ferroptosis, mitochondrial damage, or immunogenic cell death, and may also act by silencing oncogenesis-related pathways. For example, a Gboxin nanomedicine coated with hybrid cancer-cell and mitochondrial membranes exploits tumor-cell vulnerability in mitochondrial metabolism to achieve selective killing of GBM, exemplifying a design rationale for tumor mitochondrial targeting that primarily disrupts cellular function [52]. Macrophage membrane-camouflaged nanoparticles can induce ferroptosis in GBM by exacerbating mitochondrial damage, further indicating that mitochondrial targeting in tumors emphasizes activation of death signals rather than maintenance of mitochondrial functional stability [87].

In addition, bioengineered protein nanocarriers can facilitate siRNA escape from lysosomes and enable targeted RNA interference therapy for GBM. This suggests that tumor delivery must not only achieve drug accumulation in tumor tissue, but also address intracellular delivery challenges such as endosomal/lysosomal escape and cytoplasmic release. Brain-targeted antigen-generating nanoparticles can improve the prognosis of GBM by generating antigens in situ and activating immune responses, indicating that delivery systems can also enhance antitumor effects through immune activation [99]. Studies of temozolomide-penetrating delivery nanoparticles and peptide-conjugated lipid nanoparticles for siRNA delivery further indicate that formulation design for brain-tumor delivery should be optimized to enhance drug accumulation, reverse drug resistance, and improve the effects of combination therapies [129].

### 4.4. Differences in Therapeutic Windows and Administration Strategies

Inflammatory diseases typically progress through distinct stages, including acute onset, subacute repair, and chronic recurrence. Therefore, administration strategies should be adjusted according to disease progression and BBB status [114,115]. For example, during the acute stage of stroke, transient BBB opening may facilitate drug entry into brain tissue; however, excessive increases in barrier permeability may also exacerbate cerebral edema, immune-cell infiltration, and neurovascular unit injury [114,116]. Therefore, delivery systems for inflammatory diseases are more suitable for carriers that allow repeated administration, exhibit low immunogenicity, are biodegradable, and have a broad safety margin, such as extracellular vesicles, antioxidant nanozymes, and targeted mRNA/LNP systems [76,77,122,127]. When evaluating therapeutic efficacy, attention should extend beyond drug concentrations in the brain to include inflammatory cytokine levels, glial-cell status, BBB integrity, pathological protein clearance, and the extent of neurological functional recovery [121,122,127].

In GBM, administration strategies place greater emphasis on maintaining high local drug concentrations within tumors, controlling residual lesions, and combining treatment with other therapeutic modalities. Because the BBB/BBTB at tumor-infiltrative margins and postoperative residual regions may remain relatively intact, systemic administration often fails to achieve and maintain effective drug concentrations at all lesion sites [14,118]. Convection-enhanced delivery can bypass the BBB/BBTB and directly infuse drugs into the brain parenchyma, thereby increasing local drug exposure. This approach is particularly suitable for macromolecular drugs, viral vectors, and other therapeutic formulations that do not readily cross these barriers [130]. Postoperative local administration and sustained-release systems within the tumor cavity can maintain high drug concentrations in regions at high risk of recurrence and represent complementary strategies with translational potential for GBM treatment [131]. In addition, focused ultrasound combined with microbubbles can achieve local, transient, and relatively controllable BBB opening. However, the extent of barrier opening, safety, feasibility of repeated administration, and optimal timing when combined with radiotherapy or chemotherapy still require further investigation and optimization [132].

**Figure 4:**
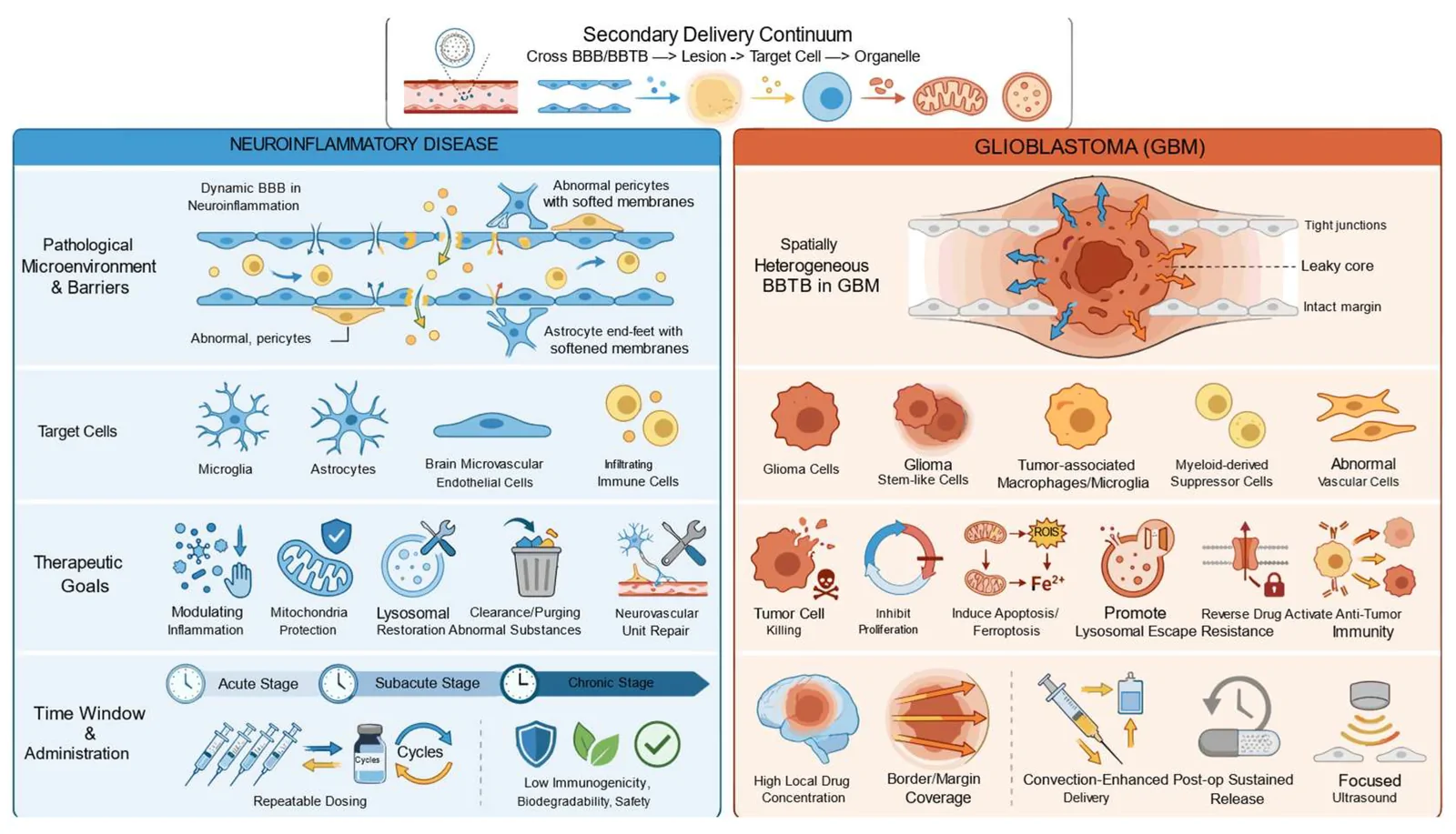
Disease-specific secondary delivery strategies for neuroinflammation and GBM. Differences in barrier status, target cells, therapeutic goals, and administration windows guide the design of organelle-targeted delivery systems. Created in BioRender. chen, B. (2026) https://BioRender.com/u65kaft.

In summary, the differences in brain delivery between inflammatory diseases and brain tumors cannot be explained solely by differences in BBB or BBTB permeability. Rather, they are jointly influenced by multiple factors, including the pathological microenvironment, target-cell types, therapeutic objectives, and timing of administration. Future brain-delivery systems may possess a degree of universality at the platform level. For example, extracellular vesicles, lipid nanoparticles, cell membrane-biomimetic nanoparticles, polymeric nanoparticles, and protein nanocarriers can all serve as basic delivery architectures. However, the design of specific functional modules should still reflect disease-specific differences. For inflammatory diseases, delivery systems should prioritize anti-inflammatory and antioxidant effects, promotion of autophagy, and mitochondrial protection. In contrast, brain-tumor delivery systems require payloads such as cytotoxic agents, nucleic acid therapeutics, immune activators, ferroptosis inducers, and drug-resistance reversal agents. Therefore, drug-delivery research for CNS diseases should move beyond the question of whether a carrier can cross biological barriers and further consider how to enter appropriate target cells at the appropriate disease stage, achieve controlled release within the appropriate subcellular organelles, and produce predictable therapeutic effects (Figure 4).

## 5. Current Technical Bottlenecks and Translational Challenges

In recent years, nanodelivery systems, liposomes, polymeric nanoparticles, biomimetic membrane-coated carriers, exosome-like carriers, and focused ultrasound-assisted delivery approaches have shown considerable research potential for the treatment of GBM. However, their clinical translation still faces multiple challenges [14,15,74,75]. For CNS tumors, delivery efficiency depends not only on whether carriers can cross the BBB, but also on whether they can adapt to the interpatient heterogeneity of the BBTB and achieve stable, predictable, and therapeutically effective drug concentrations within tumor tissue [133]. Therefore, evaluation of GBM nanodelivery systems should shift from the single criterion of “BBB-crossing ability” towards a translational framework encompassing barrier crossing, intratumoural penetration, cellular uptake, subcellular localization, safety, and manufacturability.

### 5.1. BBB/BBTB Crossing Efficiency and Interpatient Heterogeneity

The BBB and BBTB are the primary barriers limiting drug delivery for GBM. However, the BBTB is not simply a “disrupted BBB”, but a barrier determined jointly by abnormal vascular architecture, endothelial-cell transcriptional states, pericyte coverage, basement membrane remodeling, and the tumor microenvironment [133,134]. In patients with GBM, barrier integrity differs substantially among contrast-enhancing regions, necrotic regions, infiltrative margins, and relatively normal brain tissue. Therefore, even when drugs or nanoparticles enter tumor regions, heterogeneous distribution may result in insufficient drug concentrations in certain areas [133,135]. Focused ultrasound combined with microbubbles can transiently open the BBB. This approach has been used in patients with recurrent GBM to promote the entry of albumin-bound paclitaxel into tumor tissue, indicating that physical barrier-opening strategies have some potential for clinical application [136]. However, their actual delivery performance remains influenced by the extent of barrier opening, tumor vascular status, timing of administration, and interpatient variability [136,137].

Recent studies have found that extracellular particles released into peripheral blood after BBB opening may help predict GBM sensitivity to paclitaxel treatment. This indicates that barrier-modulation strategies should be integrated with imaging examinations, liquid biopsy, and pharmacokinetic measures to enable more accurate prediction of therapeutic efficacy [137]. Therefore, future evaluation of delivery systems should not rely solely on intracerebral fluorescence signals in animal models or in vitro transmembrane assays, but should establish barrier-function evaluation systems that more closely reflect clinical conditions [15,137,138].

### 5.2. Challenges of Therapeutic Delivery Across the BBB and Potential Solutions

Under physiological conditions, the BBB restricts therapeutic entry through several cooperating mechanisms. Continuous brain microvascular endothelial cells joined by tight junctions largely suppress paracellular transport, whereas transcellular diffusion favors only a limited subset of small molecules with suitable lipophilicity, charge, and hydrogen-bonding properties. Large biologics, nucleic acids, and most hydrophilic drugs therefore show negligible spontaneous penetration. Even compounds that enter endothelial cells may be returned to the circulation by efflux transporters, including P-glycoprotein and breast cancer resistance protein, or undergo enzymatic degradation before reaching the brain parenchyma [9,10,11,12]. High plasma-protein binding, rapid systemic clearance, and restricted diffusion through the brain extracellular space can further reduce the unbound drug concentration at the target site. Importantly, pathological BBB disruption does not eliminate these problems. Barrier permeability varies among patients, brain regions, and disease stages, and relatively intact vessels may coexist with severely damaged regions. Consequently, increased permeability can remain insufficient for uniform drug delivery while simultaneously increasing the risks of edema, immune-cell infiltration, and neurovascular injury [14,15,16].

One group of approaches seeks to cross the BBB without disrupting it. Medicinal-chemistry optimization and prodrug design can improve passive diffusion or exploit endogenous nutrient transporters, although changes in lipophilicity or transporter recognition may also alter systemic distribution, pharmacological selectivity, and toxicity. Carrier- and receptor-mediated transcytosis can use transport systems associated with transferrin receptors, low-density lipoprotein receptor-related protein 1, insulin receptors, or apolipoproteins to move payloads through brain endothelial cells. For these systems, ligand affinity and valency require careful optimization because overly strong binding may promote endothelial retention or lysosomal degradation rather than release into the brain. Liposomes, polymeric nanoparticles, lipid nanoparticles, ferritin-based carriers, extracellular vesicles, and cell membrane-biomimetic platforms can additionally protect unstable payloads and combine BBB-crossing ligands with lesion-, cell-, or organelle-targeting modules [10,12,15,16,80,138]. Nevertheless, protein-corona formation, uptake by the mononuclear phagocyte system, variable receptor expression, immunogenicity, and the often small fraction of a systemic dose that is successfully transcytosed remain important limitations.

A second group of approaches transiently modulates the barrier or bypasses it. Focused ultrasound combined with microbubbles can open the BBB locally and reversibly and has shown clinical feasibility for enhancing drug exposure in recurrent GBM [132,136]. Its use, however, requires control of acoustic pressure, microbubble dose, treatment volume, and administration timing to limit hemorrhage, sterile inflammation, edema, and cumulative vascular injury during repeated treatment. When systemic BBB crossing remains inadequate, intranasal, intrathecal, or intraventricular administration can partially bypass the vascular barrier, whereas convection-enhanced delivery and postoperative implants can maintain high local concentrations in selected brain regions [37,130,131]. These routes are valuable for macromolecules, viral vectors, or locally acting agents, but their broader use may be limited by invasiveness, reflux or uneven tissue distribution, procedure-related risk, and poor coverage of diffuse or multifocal disease.

From an administration-route perspective, therapeutic agents can reach the brain through systemic, nose-to-brain, cerebrospinal-fluid-based, direct parenchymal, or locoregional depot approaches. Oral administration is convenient for brain-penetrant small molecules but is limited by variable absorption, first-pass metabolism, efflux transporters, and the requirement for sufficient BBB permeability. Intravenous administration is more compatible with biologics and nanocarriers and can be combined with receptor-mediated transcytosis or focused ultrasound; however, systemic clearance, peripheral off-target exposure, and low transvascular delivery efficiency remain major constraints. Intranasal delivery may use olfactory and trigeminal pathways to bypass part of the BBB and is comparatively noninvasive, but mucociliary clearance, small dose volumes, and variable deposition can reduce reproducibility. Intrathecal or intraventricular administration directly accesses the cerebrospinal fluid (CSF) and can support macromolecular or gene-based therapeutics, although it is invasive and often provides limited penetration into the deep brain parenchyma. Direct intraparenchymal injection and convection-enhanced delivery achieve high local exposure, whereas postoperative wafers, injectable hydrogels, or implants can sustain release near a resection cavity; these approaches are better suited to focal GBM than to diffuse neuroinflammation because catheter placement, reflux, tissue injury, and incomplete lesion coverage restrict repeated or widespread use. Route selection should therefore be matched to disease distribution and treatment duration: diffuse inflammatory disorders generally favor repeatable systemic or intranasal delivery, whereas localized or recurrent GBM may justify convection-enhanced delivery or intracavitary depots when the expected gain in tumor exposure outweighs procedural risk [4,5,6,107,130,131,132].

No single strategy can therefore resolve every BBB-related delivery problem. The preferred approach should be matched to payload properties, disease stage, BBB or BBTB integrity, lesion distribution, target-cell identity, and the required duration of exposure. A staged system may, for example, combine a circulation-stable carrier with controlled transcytosis or image-guided barrier opening, followed by lesion-responsive release and cell- or organelle-specific delivery. Such added complexity is justified only when each step produces a measurable gain. Accordingly, preclinical evaluation should quantify unbound drug exposure, regional and cellular biodistribution, barrier recovery, and pharmacodynamic effects rather than relying solely on whole-brain fluorescence or total tissue concentration. Human BBB-on-a-chip systems, vascularized organoids, patient-derived models, pharmacokinetic analysis, and noninvasive imaging may help identify patients and dosing windows in which a particular strategy is both effective and acceptably safe [15,137,138].

### 5.3. Organelle Off-Targeting and Nonspecific Accumulation

Crossing the BBB/BBTB is only the first step in brain-tumor delivery. Carriers must further achieve tumor tissue penetration, cellular uptake, endosomal escape, and localization to specific subcellular organelles before drugs can reach their true sites of action and exert therapeutic effects [52,139]. In recent years, mitochondria-targeted nanodelivery systems have received considerable attention. For example, TPP-modified chitosan nanoparticles can promote the mitochondrial accumulation of temozolomide, whereas mitochondria-targeted nanosystems capable of generating ROS can enhance antitumor activity by increasing oxidative stress [139]. In addition, cancer cell–mitochondria hybrid membrane-coated nanoparticles loaded with Gboxin can improve stability during systemic circulation and enhance tumor-cell uptake and mitochondrial localization, providing experimental evidence for biomimetic membrane-assisted subcellular organelle delivery [52].

However, it should be noted that lipophilic cations such as TPP primarily rely on membrane potential-driven accumulation. In addition to mitochondria, they may exhibit nonspecific distribution in other organelles with membrane potential-related characteristics or in highly metabolically active normal cells, thereby increasing the risk of off-target toxicity. Therefore, although multistage targeting strategies can improve therapeutic efficacy, they also increase the complexity of carrier design, uncertainty in in vivo behavior, and the difficulty of safety evaluation.

More importantly, unintended uptake by healthy neurons, astrocytes, microglia, brain endothelial cells, or peripheral organs may convert an intended organelle-directed mechanism into collateral toxicity. In normal cells, excessive mitochondrial accumulation of cationic or ROS-generating constructs may depolarize mitochondrial membranes, impair oxidative phosphorylation and ATP production, disrupt calcium and redox homeostasis, and trigger cytochrome c-dependent apoptosis or other regulated cell-death pathways [24,25]. Likewise, strong lysosomal retention or membrane-disruptive escape may alter lysosomal acidification, block autophagic flux, cause lysosomal membrane permeabilization and cathepsin release, and activate inflammasome-associated inflammation or cell death [26,27,28,103]. These risks are particularly relevant in the CNS because post-mitotic neurons have limited regenerative capacity, whereas injury to endothelial cells, astrocytes, or microglia may compromise the neurovascular unit or amplify neuroinflammation.

Risk mitigation should therefore be incorporated into both carrier design and preclinical evaluation. Design options include hierarchical or AND-gated targeting that combines lesion- and cell-specific recognition with organelle-targeting motifs; shielding or using cleavable cationic groups that are exposed only in acidic, enzymatic, hypoxic, or ROS-rich diseased microenvironments; optimizing ligand density, particle size, surface charge, dose, and dosing interval; favoring biodegradable carriers and triggered payload release; and using local or image-guided administration where appropriate [140,141,142,143,144]. Safety studies should quantify biodistribution at the organ, cell, and organelle levels and compare diseased with healthy tissues after both single and repeated dosing. In addition to routine histology and serum chemistry, relevant endpoints include mitochondrial membrane potential, respiration, ATP, mitochondrial ROS, and cytochrome c release, together with lysosomal pH, membrane integrity, cathepsin leakage, and autophagic flux. These measurements should be integrated with neurobehavioral testing, glial and endothelial responses, reversibility after treatment withdrawal, and studies in primary human cells, BBB models, and patient-derived organoids to define an organelle-specific therapeutic window before clinical translation [80,81,82,145,146,147,148,149].

### 5.4. Carrier Biocompatibility and Long-Term Toxicity

Patients with GBM often require repeated administration or combination therapy. Therefore, safety evaluation of nanocarriers cannot be limited to short-term cytotoxicity and acute toxicity in animal models [74,145]. For CNS delivery systems, brain retention, glial-cell activation, complement responses, neuroinflammation, remodeling of the immune microenvironment, and long-term clearance pathways may all affect clinical safety [80,81,145]. Biomimetic membrane-coated carriers, cell membrane nanoparticles, and exosome-like delivery systems offer certain advantages in immune evasion and homotypic targeting. However, differences among source materials, including membrane protein composition, potential immunogenicity, and batch-to-batch product consistency, remain key issues in pharmaceutical research and quality evaluation [80,82].

Meanwhile, some emerging nanoplatforms can enhance anti-GBM effects by inducing ferroptosis, cuproptosis, autophagy, or immune activation. However, within the complex brain-tumor microenvironment, these effects may also exacerbate inflammatory responses or damage surrounding normal cells [81,88,146]. Therefore, preclinical safety assessment of GBM nanomedicines should be more comprehensive. In addition to conventional toxicity measures, it should include neurobehavioral performance, drug distribution across different brain regions, changes in inflammatory cytokines, glial-cell responses, and long-term tissue accumulation.

Several studies have included repeated administration, but these were generally short efficacy studies rather than dedicated chronic toxicology programs. For example, Zou et al. administered the mitochondria-targeted hybrid cancer cell–mitochondrial membrane-coated Gboxin-loaded nanoparticles (HM-NPs@G) formulation intravenously every 3 days for a total of five doses and reported stable body weight and no evident histopathological injury in major organs in the nanoparticle-treated groups [53]. In a 1-methyl-4-phenyl-1,2,3,6-tetrahydropyridine (MPTP) -induced Parkinson’s disease model, Zheng et al. applied two treatment sessions of mitochondria-targeted Cu_2−_xSe/curcumin nanoparticles coated with triphenylphosphonium-modified macrophage membranes (CSCCT nanoparticles) and observed no obvious damage in hematoxylin and eosin (H&E) -stained major organs [77]. These findings provide preliminary repeat-dose tolerability signals, but they do not establish chronic safety because exposure lasted only days to a few weeks and post-treatment recovery, cumulative organelle dysfunction, immunogenicity, and delayed neurotoxicity were not comprehensively examined. Within the CNS studies summarized here, convincing long-duration data for repeated exposure to lysosome-, nucleus-, endoplasmic reticulum-, or Golgi-targeted carriers remain particularly scarce.

Future safety programs should therefore match the exposure duration and route to the intended clinical regimen and include vehicle- and ligand-matched controls, multiple dose levels, and recovery groups. Serial toxicokinetic and biodistribution analyses should determine whether the carrier, targeting ligand, degradation products, or payload accumulate in different brain regions, healthy CNS cell types, and peripheral organs after each treatment cycle. Repeated-dose assessment should combine hematology, serum chemistry, complement activation, anti-carrier antibodies, cytokine profiling, organ histopathology, and neurobehavioral testing with cell-type-resolved measurements of glial and endothelial responses and direct assays of mitochondrial and lysosomal function. Follow-up after treatment withdrawal is essential to establish reversibility and to determine whether repeated exposure causes accelerated clearance, loss of targeting efficacy, or cumulative organelle injury. Studies in aged, immunocompetent animals and, where justified, larger species would further improve translational relevance [74,80,81,82,145].

### 5.5. Clinical-Trial Landscape of Organelle-Targeted Delivery

No registered interventional trial was identified in CNS inflammation or brain tumors in which a liposome, polymeric nanoparticle, extracellular vesicle, or other nanocarrier was explicitly engineered to deliver a payload to mitochondria, lysosomes, the endoplasmic reticulum, the Golgi apparatus, or the nucleus. Thus, the disease-matched nanoplatforms discussed above remain at the preclinical stage.

Nevertheless, several clinically advanced technologies provide important translational precedents. MitoQ, a triphenylphosphonium-conjugated antioxidant that accumulates in mitochondria, completed a phase II trial in Parkinson’s disease, but did not slow disease progression [150]. Elamipretide, an aromatic-cationic peptide that localizes to the inner mitochondrial membrane, underwent phase II and long-term studies and received accelerated approval from the U.S. Food and Drug Administration (FDA) for Barth syndrome in 2025 [151]. The clearest delivery-vector precedent is tividenofusp alfa-eknm, in which iduronate-2-sulfatase is fused to a transferrin-receptor-binding Fc transport vehicle: it crosses the BBB by receptor-mediated transcytosis and is subsequently internalized through the mannose-6-phosphate receptor and trafficked to lysosomes. Following phase I/II and phase II/III evaluation, it received accelerated FDA approval for neurologic manifestations of Hunter syndrome in 2026 [152]. These examples validate organelle-localizing small molecules/peptides and biologic shuttles, but not yet multifunctional nanocarriers for GBM or neuroinflammation.

### 5.6. Translational Gap Between Preclinical Models and Human Pathology

Most GBM nanomedicines are currently evaluated primarily in subcutaneous tumor models, orthotopic transplantation tumor models, or immunodeficient mouse models. However, these models cannot fully recapitulate the infiltrative growth, BBTB heterogeneity, immunosuppressive microenvironment, and post-treatment recurrence observed in patient tumors [147,148]. Although orthotopic tumor models provide a growth environment that is relatively closer to that in the brain, they remain limited with respect to human vascular formation, immune-cell interactions, and patient-specific genetic backgrounds, which may lead to overestimation of delivery efficiency and antitumor efficacy.

In recent years, BBB organoids, vascularized brain organoids, and patient-derived GBM organoids have provided new tools for evaluating drug delivery across barriers, tumor tissue penetration, and individualized therapeutic responses [148,149]. However, these models still have limitations, including insufficient blood flow shear stress, absence of an immune system, limited stability during long-term culture, and inadequate simulation of pharmacokinetics. Therefore, evaluation systems with greater clinical translational value should integrate information from patient-derived organoids, BBB-on-a-chip platforms, orthotopic patient-derived xenograft (PDX) models, imaging tracers, and liquid biopsy to establish a more comprehensive preclinical evidence chain across multiple levels.

## 6. Future Directions

In view of the current limitations of brain-targeted nanodelivery in crossing the BBB, lesion accumulation, subcellular organelle localization, and validation of off-target effects, future research should gradually shift from single-targeting strategies towards progressive precision-delivery systems that coordinate actions across “multiple barriers, tissues, and subcellular organelles” [14,140]. In brain tumors, delivery systems must not only cross the BBB/BTB, but also penetrate heterogeneous tumor tissues and achieve intracellular accumulation at sites such as mitochondria, lysosomes, or the nucleus [14,141]. In contrast, delivery for inflammatory brain diseases should place greater emphasis on dynamically responding to injured vasculature, immune-cell infiltration, microglial polarization, and changes in the inflammatory microenvironment. Therefore, its delivery objectives should not simply adopt the tumor-treatment paradigm of “killing tumor cells and prolonging local retention”, but should instead be specifically designed according to the characteristics of inflammatory processes [122,153].

First, multiorganelle coordination and cascade targeting will become important directions for optimizing brain-delivery systems. Conventional BBB receptor-mediated delivery commonly relies on barrier-crossing ligands such as transferrin, apolipoprotein E (ApoE), or rabies virus glycoprotein (RVG) peptides. However, single-ligand strategies often cannot simultaneously meet the requirements of multiple sequential steps, including stability during blood circulation, BBB crossing, lesion recognition, and intracellular drug release [140,141]. Future strategies may therefore employ dual-ligand or multi-ligand cascade designs. For example, an ApoE protein corona or BBB-crossing ligand could first facilitate carrier entry into brain tissue, followed by the incorporation of tumor-cell recognition ligands, mitochondrial-targeting moieties, or lysosomal escape modules to enable more specific intracellular delivery [154].

Recent studies have shown that nanosystems with both BBB-penetrating and mitochondrial-targeting capabilities can further enhance therapeutic effects by disrupting mitochondrial electron transport in GBM cells or inducing ROS generation. Compared with simply increasing drug concentrations in the brain, the strategy of “BBB crossing plus organelle intervention” may therefore provide more profound therapeutic effects [155,156]. For nucleic acid therapeutics, efficient escape from lysosomes is likewise a key determinant of therapeutic efficacy. Studies have shown that engineered protein nanocarriers can enhance RNA interference therapy against GBM after promoting lysosomal escape of siRNA [99].

Second, visualization and tracking technologies should evolve from merely “demonstrating carrier entry into brain tissue” to “real-time verification of carrier accumulation within lesions and specific subcellular organelles” [142]. At present, many studies still use ex vivo fluorescence, tissue sections, or endpoint drug concentrations as evidence of delivery, making it difficult to determine whether nanocarriers have truly completed BBB crossing, lesion-specific release, and organelle localization [142,143]. Near-infrared-II (NIR-II) imaging, magnetic resonance imaging (MRI), fluorescence resonance energy transfer (FRET), and theranostic probes may provide solutions to this challenge. For example, NIR-II-engineered exosome probes have been used for imaging-guided therapy of orthotopic GBM, indicating that deep-penetration imaging technologies can facilitate monitoring of carrier distribution in the brain and inform the selection of therapeutic timing [142]. MRI-guided functionalized nanoliposomes and galactosylated ferrite platforms have demonstrated the potential to integrate lesion localization, drug delivery, and therapeutic-response feedback, particularly for ferroptosis and immunomodulatory therapies requiring precise spatial control [143]. In addition, in inflammatory models, FRET has been used to monitor in real time nanocarrier release at stroke microthrombi and the process of BBB crossing, providing a reference for addressing challenges such as difficult-to-verify off-target effects and nonvisualized drug-release processes [144].

Finally, differentiated and personalized delivery strategies for inflammation and tumors should become a central direction for the field. In tumors, delivery systems should prioritize the challenges of tumor heterogeneity, the immunosuppressive microenvironment, deep infiltration, and amplification of subcellular organelle-mediated death signals. In inflammatory brain diseases, delivery strategies should instead focus on BBB repair, regulation of immune-cell function, attenuation of oxidative stress, and remodeling of the neurovascular unit. Therefore, future brain-targeted nanomedicines should not pursue a single, universal delivery platform. Rather, they should integrate disease stage, BBB integrity, inflammatory status, relevant cellular composition, and specific therapeutic targets to develop more tailored delivery strategies. This shift will advance brain-delivery research from the question of “whether a carrier can enter brain tissue” towards a precision-treatment paradigm that addresses “when it should enter the brain, which cells it should act on, to which organelles it should localize, and whether it can produce verifiable therapeutic effects.”

## 7. Conclusions

Secondary delivery strategies, extending from brain targeting to organelle targeting, provide a new paradigm for the treatment of CNS inflammation and brain tumors. However, current delivery systems remain unable to achieve the complete sequential process of BBB penetration, lesion accumulation, cellular uptake, and organelle localization. Owing to their functionalizable surfaces and barrier-crossing capabilities, nanocarriers have demonstrated considerable potential to traverse the BBB and BBTB in multiple CNS disease models. Nevertheless, crossing these barriers is only the first step. To achieve second-stage delivery, carriers must efficiently escape from endosomal/lysosomal compartments and precisely reach the actual sites of action, including mitochondria, lysosomes, or the nucleus. At present, most studies remain limited to demonstrating enhanced brain distribution or cellular uptake. However, merely increasing the total drug concentration in the brain is insufficient to ensure therapeutic concentrations at the actual pharmacological targets. This is a key reason why many candidate drugs that show promising activity in preclinical studies ultimately fail to translate into clinical benefit. Encouragingly, recent breakthroughs in cascade-targeting design, in vivo visualized tracking, and disease-stratified drug administration have advanced two-stage, organelle-targeted delivery from a conceptual framework toward functional validation. This framework is expected to enable a complete cascade from barrier penetration to organelle intervention for the precision treatment of CNS diseases.

## Figures and Tables

**Table 1:** Representative brain-targeted and organelle-targeted nanodelivery studies.

Target	Disease Model	Delivery Platform	Key Sequential Targeting Features and Therapeutic Outcomes	Reference
Mitochondria	Glioma	Glucose/TPP^+^ co-modified redox-responsive liposomes [doxorubicin (Dox)/lonidamine (LND)]	Glucose promotes tumor-cell uptake, whereas TPP^+^ promotes mitochondrial accumulation, enabling synergistic metabolic therapy.	[72]
Mitochondria	Parkinson’s disease	TPP-modified macrophage membrane-coated Cu_2−_xSe/curcumin nanoparticles	Respond to an inflammatory microenvironment and localize to mitochondria in injured neurons, promoting mitochondrial biogenesis.	[76]
Mitochondria	Neurodegenerative diseases	Macrophage-derived nanovesicles delivering ursodeoxycholic acid	Inflammation tropism enables BBB crossing and delivery to neuronal mitochondria, alleviating mitochondrial dysfunction.	[77]
Mitochondria	Cerebral ischemia–reperfusion injury	Tannic acid/melanin-modified polyoxometalate nanomedicine	Recognizes matrix proteins exposed at the injured BBB and targets neuronal outer mitochondrial membranes, limiting mitochondrial damage.	[86]
Mitochondria	GBM	Gboxin nanomedicine coated with hybrid cancer-cell/mitochondrial membranes	Enables BBB penetration, homologous tumor-cell uptake, and mitochondrial targeting, thereby inhibiting oxidative phosphorylation.	[52]
Mitochondria	GBM	Angiopep-2-modified macrophage membrane-camouflaged saALOX15 nanoparticles	Crosses the BBB and induces mitochondrial damage-related ferroptosis, improving radiosensitivity.	[87]
Nucleus (functional delivery)	GBM	Angiopep-2-modified lipid-polymer nanoparticles delivering Cas9/sgRNA	Achieves STAT3 gene editing after BBB crossing, remodeling tumor vasculature and the immune microenvironment.	[73]
Endosome/lysosome (escape)	GBM	Engineered protein nanocarriers delivering siRNA	Facilitates lysosomal escape of siRNA into the cytosol and enhances RNA-interference therapy.	[99]

## Data Availability

No new data were created or analyzed in this study. Data sharing is not applicable to this article.
